# Dietary acid load and its interaction with CETP TaqB1 polymorphisms on lipid profile among patients with Type 2 diabetes mellitus

**DOI:** 10.1186/s12902-023-01391-6

**Published:** 2023-07-05

**Authors:** Faezeh Abaj, Zahra Esmaeily, Zeinab Naeini, Ehsan Alvandi, Masoumeh Rafiee, Fariba Koohdani

**Affiliations:** 1 Department of Community Nutrition, School of Nutritional Sciences and Dietetics, Tehran University of Medical Sciences, Tehran, Iran; 2 Department of Cellular and Molecular Nutrition, School of Nutritional Sciences and Dietetics, Tehran University of Medical Sciences, Tehran, Iran; 3 School of Medicine, Western Sydney University, Campbelltown, NSW Australia; 4 Department of Clinical Nutrition, School of Nutrition and Food Science, Isfahan University of Medical Sciences (IUMS), Isfahan, Iran

**Keywords:** CETP polymorphism, T2DM, Nutrigenetic, Dietary acid load

## Abstract

**Objective:**

Gene-diet interaction plays a key role in the inter-individual differences in lipid abnormalities as a major risk factor for cardiovascular diseases (CVDs). Thus, we explored the interaction between CETP TaqB1 polymorphism with dietary acid load (DAL) on lipid profile among type 2 diabetes mellitus (T2DM).

**Method:**

This cross-sectional study conducted on 220 Iranian patients with T2DM. Dietary acid load (PRAL and NEAP) was calculated via a validated food-frequency questionnaire (FFQ). The polymerase chain reaction (PCR) used for genotyping Taq1B polymorphism. Biochemical markers were measured by standard protocol. The interaction between CETP Taq1B polymorphism and DAL (PRAL and NEAP) on lipid profile was performed by a generalized linear regression model (GLM).

**Results:**

The overall prevalence of rs708272 genotypes was 8.6%, 72.7% and 18.6% for B1B1, B1B2 and B2B2 genotype respectively. This study showed that people with the B1B1 genotype had greater LDL, TC, LDL/HDL, and TG when they consumed diets that scored higher on the NEAP and PRAL indexes than those with the B1B2 and B2B2 genotypes. Besides, carriers of the B1B1 allele who were in the highest tertile of NEAP, had lower HDL (P Interaction < 0.05).

**Conclusions:**

In summary, the lipid profile might be improved in B1B1 homozygotes by less adherence to DAL indexes, however, the findings should be validated in high-quality interventional studies.

## Introduction

Type 2 diabetes mellitus (T2DM) and cardiovascular diseases (CVDs) are known as common metabolic and multifactorial disorders which caused by a complex interaction between environmental factors, lifestyle and genetics [1, 2]. According to health forecasts, the global prevalence of diabetes will rise to 642 million by 2040  [3]. T2DM and its complications are recognized as major causes of mortality [4]. Over 1 million deaths per year directly caused by diabetes make it as the ninth leading cause of mortality [5]. Dyslipidemia, significantly contributes to the development of diabetes major complications including atherosclerosis and CVDs [6]. Thus, control of lipid abnormalities is of paramount importance in T2DM. Lipid profile can be modified by multiple factors includes genetic factors and environmental and their interactions as well [7]. Based on cost estimates, the expenditure of medical care for diabetes is at least 3.2 times larger than the average per capita healthcare cost which rises to 9.4 times when its complications occur [8].

Environmental factors contribute to the regulation of plasma level of lipoproteins as well as genetics [9]. Cholesteryl ester transfer protein (CETP) is one of those major factors which contributes to the metabolism of lipids via transferring cholesterol esters and triglycerides between very-low-density lipoprotein (VLDL), low-density lipoprotein cholesterol (LDL-C), and high-density lipoprotein cholesterol (HDL-C) and increases atherogenicity [10]. This action leads to the reverse transportation of cholesterol from peripheral tissues to the liver and facilitating the CE transfer from HDL3 to HDL2 which may alter the susceptibility to atherosclerosis and CVDs [11]. Studies indicated that different polymorphisms of human CETP gene are associated with its concentration and activity and also elevated level of serum lipids [12, 13]. One notable polymorphism is Taq1B (rs708272, 279th nucleotide in the first intron) which is thought to play a role in the regulation of serum lipids [14]. The less common B2 allele (the restriction site is absent) has a reverse relationship with CETP concentration and its activity and an association with higher HDL-C compared to B1 allele [15]. Most of previous studies showed that individuals with B2B2 genotype had reduced CETP and elevated HDL levels [16, 17]. In contrast, there are a few researches which found a relationship between B1B1 genotype and lower HDL-C, enhanced C-reactive protein (CRP), and the development of cardiovascular disease [18, 19]. It is taught that environmental factors including physical activity, Body mass index (BMI), and diet may explain these controversial results [20]. Both animal and human studies have shown that high fat intake especially high saturated fat increases CETP activity and HDL-C level [21–24]. findings highlight the fact that even 1% increase in HDL-C level causes 2–3% reduction in CVDs [25]. Tricia Y Li et al. found a relationship between lower carbohydrate consumption and higher HDL-C concentration among B2 allele carriers [26]. However, Aitken et al. did not found noticable association [27].

Dietary acid load (DAL), determined by the potential renal acid load (PRAL) and net endogenous acid production (NEAP), reflects the dietary pattern acidity [28]. High consumption of animal protein, processed meat, and sweetened beverages and low consumption of fresh fruit and vegetables, known as western dietary pattern, lead to metabolic acidosis status and cardiometabolic abnormalities among diabetic patients [29]. Previous studies suggested a close link between DAL and lipid profile. Some of them considered hypertriglyceridemia as a major result of high DAL score [28, 30] while others showed a decrease in HDL-C level and increase in BMI level among individuals with higher tertiles of PRAL/NEAP [29, 31, 32].

Although different studies were conducted to find the impact of dietary intake on CETP Taq1B polymorphism and lipid profile, additional studies are needed to explore these findings and other gene-diet interactions [26]. We aimed to investigate the effect of CETP Taq1B polymorphism and dietary acid load determined by the Potential renal acid load (PRAL) and net endogenous acid production (NEAP) interaction in relation to lipid abnormalities among patients with T2DM.

## Method and material

### Participants

The current study is part of a larger cross-sectional research on 220 patients with T2DM Were recruited from the Tehran diabetes centers from individuals with fasting blood sugar (FBS) levels ≥ 126 mg/dl or consuming glucose-lowering medicines [33]. For this study we calculated minimum sample size in our first project [14] based on minor allele frequency of the CETP Taq1Bpolymorphism in the world and Iranian population.

Patients who were under 35 or over 65 years old, insulin-using, pregnant or lactating women were excluded. All participants gave their written informed consent and the ethical principles established in the Declaration of Helsinki and the protocol of study approved by the ethics committee of Tehran University of Medical Sciences (TUMS) (no.15060).

### General information and physical activity assessment

All information about age, sex, job, smoking, medical information was collected using interview. Anthropometric information including physical activity (METs), waist circumference (WC), and body mass index (BMI) were measured according to standard protocols. Their weight was taken without shoes but with light clothes on. BMI was estimated as weight (kg) divided by height2 (m2). The narrowest part of the abdomen and the midway between the lowest rib and the iliac crest were used to quantify WC. Daily physical activity was assessed by the classified metabolic equivalent task (MET) questionnaire, which was validated in Iran by Kelishadi et al. [34].

### Dietary assessment

Data on dietary consumption were gathered using a validated 147-item semi-quantitative food frequency questionnaire (FFQ) [35]. The PRAL and NEAP were used to compute DAL. The PRAL was calculated using the method recommended by Remer et al. as follows:. [36]

PRAL (mEq/d) is equal to 0.4888 g/day of protein plus 0.0366 mg/day of phosphorus, 0.205 mg/day of potassium, 0.0125 mg/day of calcium, and 0.0263 mg/day of magnesium.

Moreover, Frassetto et al.'s NEAP formula was as follows: [37]

NEAP (mEq/d) is equal to [54.5 potassium intake (mEq/d) protein intake (g/d)].—10.2

The categorized metabolic equivalent task (MET) questionnaire, which was validated in Iran by Kelishadi et al., was used to measure daily physical activity [38].

### Biochemical evaluation and genotyping

To measure serum lipids, whole blood samples were collected following a 10- to 12-h fast. Enzymatic approach was used to evaluate the levels of triglycerides (TG) and total cholesterol (TC) in Pars Azmoon, Iran. Turbidimetry was used using a Roche Hitachi analyzer (Roche, Germany) to compute LDL-C and HDL-C. The levels of 8-isoprostane F2 (PGF2) and other inflammatory indicators, such as CRP, Interleukin-18 (IL-18), and Pentraxin 3 (PTX3), were also evaluated using the ELISA technique (Bioassay Technology Co, Germany and Shanghai Crystal Day Biotech Co., Ltd). Superoxide dismutase (SOD) serum enzymatic activity was assessed using colorimetric techniques, and total antioxidant capacity (TAC) was calculated using spectrophotometric techniques (Cayman Chemical Company, USA).

Whole blood was used to obtain genomic DNA using the salting-out extraction technique. Using a modified salting-out technique, genomic DNA was extracted from peripheral blood cells of ethylenediaminetetraacetic acid-anticogulated samples. Briefly stated, 500 ml of each blood sample were mixed with 1 mL of RBC-lysis buffer (0.32 M sucrose, 10 mM Tris–HCl, pH 8.2, 5 mM MgCl2, and 1% v/v Triton X-100), which was then incubated on ice for 15 min before centrifuging at 10,000 rpm for 2 min. After discarding the supernatant, the same procedure was repeated twice before addition of 300 ml of WBC-lysis buffer (0.45 M NaCl, 10 mM Tris–HCl, pH 8.2, and 2 mM ethylenediaminetetraacetic acid), 20 ml of 10% sodium dodecyl sulfate, and 10 ml of proteinase K (20 mg/mL), followed by incubation at 558C for 2 h. By adding 100 ml of 5 M NaCl and isopropanol (1v/v), gDNA was precipitated by mixing 100 ml of 5 M NaCl with 1v/v isopropanol. Following its removal using a thin glass rod, it was cleaned with 70% EtOH before being dissolved in 1TE buffer [39]. The CETP Taq1B polymorphism was genotyped by polymerase chain reaction (PCR) using primers (Forward: 50- CAC TAG CCC AGA GAG AGG AGTG-30 and Reverse: 50-TGA GCC CAG CCG CAC ACT AAC-30) and 8% polyacrylamide gel electrophoresis.

### Statistical analysis

The normality of data was analyzed by the Kolmogorov–Smirnov test. The sample size was calculated given a type I error of α = 0.05 and type II error of β = 80%. By considering the median amount of PRAL and NEAP, participants were divided into 3 tertiles for evaluating their adherence to these indexes. One-way ANOVA was used for the comparison of the mean difference between three genotypes (B1B1, B1B2, and B2B2) groups. Moreover, a generalized linear regression model (GLM) was used for finding the interaction between CETP Taq1B polymorphism and DAL (PRAL and NEAP) on important risk factors (BMI, WC, HDL, LDL, LDL/HDL, TC, TG, CRP, IL-18, TAC, SOD, and PGF2α) in crude and adjusted models (age, physical activity, sex, smoking, alcohol and energy intake).

## Result

In this cross-sectional investigation, associations between cardio-metabolic markers and the CETP Taq1B polymorphism were examined in 220 patients with T2DM. The overall prevalence of rs708272 genotypes was 8.6%, 72.7% and 18.6% for B1B1, B1B2 and B2B2 genotypes respectively. The genotype distributions were within HWE (*P*-value > 0.05).

### Relationship between Cardio-metabolic Markers and NEAP and PRAL

Table 1 shows the basic data on diabetic individuals in the NEAP and PRAL groups. According to their NEAP and PRAL ranking, all patients were separated into three groups. The overall energy intake of patients in the third tertile of PRAL was greater (*p* = 0.01). Moreover, estimated energy intake (EER) has shown higher between men with higher NEAP (*p* = 0.02) and PRAL (*p* = 0.005). Patients in the third tertile of PRAL had a greater tendency of consuming more carbohydrates (*P* = 0.005). Diabetic patients who were in the last tertile of NEAP (*p* < 0.001) and PRAL (*p* = 0.001) consumed higher protein. Also, higher NEAP intake was associated with higher cholesterol consumption (*p* = 0.01). Other fundamental features and biological indicators were not significantly correlated between the NEAP and PRAL groups.

**Table 1:** The association between metabolic markers with NEAP and PRAL in T2DM patients

**Tertile of NEAP**					**Tertile of PRAL**			
	**T1**	**T2**	**T3**	***P***^***a***^	**T1**	**T2**	**T3**	***P***^***a***^
**N**								
**Age (year)**	52.27 ± 6.12	52.76 ± 6.38	51.85 ± 6.73	0.69	52.52 ± 5.63	52.23 ± 6.69	51.96 ± 6.98	0.87
**BMI (kg/m2)**	29.23 ± 4.97	28.82 ± 4.82	29.45 ± 5.23	0.74	29.23 ± 5.13	28.47 ± 4.78	29.88 ± 5.04	0.23
**WC (cm)**	91.02 ± 13.10	90.74 ± 11.50	92.97 ± 11.51	0.47	90.65 ± 11.60	90.31 ± 12.90	93.93 ± 11.34	0.13
**Physical activity (MET min/week)**	38.26 ± 5.29	38.42 ± 5.84	37.69 ± 4.75	0.68	38.95 ± 6.70	37.57 ± 4.23	37.91 ± 4.58	0.25
**Energy intake (kcal/day)**	2415.95 ± 821.84	2519.88 ± 933.89	2730.22 ± 906.50	0.09	2681.02 ± 1032.46	2312.04 ± 697.54	2680.92 ± 883.14	**0.01**
**Protein (g/d)**	78.19 ± 24.11	87.98 ± 32.43	101.54 ± 36.29	** < 0.001**	88.63 ± 34.22	79.28 ± 22.35	98.89 ± 38.35	**0.001**
**Carbohydrate (g/d)**	329.46 ± 115.02	334.18 ± 128.80	355.72 ± 125.03	0.38	366.29 ± 144.14	303.64 ± 87.12	345.58 ± 129.92	**0.008**
**Total Fat (g/d)**	96.43 ± 44.71	101.43 ± 53.90	107.73 ± 45.60	0.36	106.23 ± 52.96	94.36 ± 47.74	103.79 ± 44.57	0.29
**Cholesterol (g/d)**	187.92 ± 84.48	220.84 ± 98.87	269.23 ± 251.34	**0.01**	207.80 ± 108.35	208.68 ± 79.10	259.30 ± 254.76	0.10
**EER.(Men)**	2501.74 ± 220.08	2572.74 ± 265.71	2698.64 ± 314.03	**0.02**	2579.35 ± 269.35	2500.15 ± 237.92	2726.28 ± 300.97	**0.005**
**EER.(Women)**	2033.89 ± 177.86	2008.71 ± 188.05	2069.51 ± 221.80	0.37	2023.61 ± 173.04	2017.45 ± 184.40	2080.22 ± 231.29	0.27
**HDL-c(mg/dl)**	54.98 ± 11.96	52.66 ± 11.04	52.43 ± 11.09	0.32	54.19 ± 11.83	53.33 ± 11.41	52.42 ± 10.94	0.64
**LDL-c(mg/dl)**	121.02 ± 39.61	121.12 ± 34.28	113.41 ± 27.46	0.29	122.65 ± 42.11	119.13 ± 30.53	113.80 ± 27.85	0.28
**CH(mg/dl)**	204.70 ± 66.18	219.36 ± 91.72	209.04 ± 72.06	0.50	207.48 ± 64.12	213.27 ± 89.74	211.64 ± 76.03	0.89
**LDL/HDL**	2.25 ± 0.73	2.35 ± 0.67	4.73 ± 21.36	0.39	2.32 ± 0.80	2.29 ± 0.59	4.77 ± 21.51	0.38
**TG(mg/dl)**	180.35 ± 121.23	186.57 ± 118.90	120.61 ± 104.36	0.94	187.91 ± 120.96	174.93 ± 116.76	187.84 ± 122.70	0.75
**Leptin(ng/ml)**	24.62 ± 16.38	25.19 ± 15.53	19.90 ± 14.96	0.54	25.54 ± 16.94	23.31 ± 13.97	22.12 ± 16.97	0.76
**Ghrelin(ng/ml)**	2.05 ± 0.93	2.08 ± 0.92	2.76 ± 1.46	0.11	2.04 ± 0.89	2.09 ± 0.98	2.71 ± 1.42	0.13
**CRP (mg/L)**	2.91 ± 1.32	2.13 ± 1.76	2.70 ± 1.21	0.21	2.96 ± 1.48	2.16 ± 1.60	2.80 ± 1.19	0.17
**PTX3(ng/ml)**	2.67 ± 0.36	2.73 ± 0.44	2.79 ± 0.59	0.72	2.58 ± 0.41	2.75 ± 0.40	2.84 ± 0.53	0.23
**IL18(pg/ml)**	259.94 ± 34.91	242.31 ± 27.45	241.85 ± 20.56	0.09	248.14 ± 34.87	249.98 ± 30.08	241.41 ± 21.52	0.66
**TAC(g/dl)**	2.38 ± 0.38	2.56 ± 0.61	2.41 ± 0.60	0.55	2.37 ± 0.46	2.54 ± 0.56	2.40 ± 0.59	0.54
**SOD(U/ml)**	0.13 ± 0.03	0.15 ± 0.05	0.14 ± 0.03	0.53	0.14 ± 0.03	0.14 ± 0.05	0.14 ± 0.03	0.85
**PGF2α(pg/ml)**	73.18 ± 7.44	74.56 ± 5.68	70.93 ± 7.61	0.27	73.61 ± 8.03	73.55 ± 5.60	71.73 ± 7.62	0.67

Data are presented as mean ± standard deviation (SD)

*Abbreviation: PRAL* Potential renal acid load, NEAP Net endogenous acid production, *BMI* Body mass index, *WC* Waist circumference, *HDL-c* High density lipoprotein cholesterol, *LDL-c* Low density lipoprotein cholesterol, *CH* Cholesterol, *TG* Triglyceride, *CRP* C-reactive protein, *PTX3* Pentraxin 3, *IL18* Interleukin 18, *TAC* Total antioxidant capacity, *SOD* Superoxide dismutase, *PGF2α* Prostaglandinf2α

^a^Obtained from ANOVA

### NEAP and PRAL interactions with the CETP Taq1B polymorphism and their effects on lipid profiles

Tables 1, 2 and 3 show the relationship between the CETP Taq1B polymorphism and the tertiles of the NEAP and PRAL scores on lipid profiles.

**Table 2:** The interaction between CETP Taq1B polymorphism and NEAP on cardiovascular risk factors

**Variables**	**NEAP**	**Mean±SD**			**P**_**1**_	**P**_**2**_	
		**B1B1**	**B1B2**	**B2B2**	**Model (1)**	**Model (2)**	**P -trend**
**LDL (mg/dl)**	**T**_**1**_	106.37±11.8	121.94±4.67	126.07±8.92	**0.01**	**0.01**	**0.03**
	**T**_**2**_	119.71±12.61	121.19±4.86	121.5±7.86			
	**T**_**3**_	146.75±16.69	112.59±4.23	103.12±11.8			
**HDL (mg/dl)**	**T**_**1**_	64.25±5.48	53.52±1.53	58.64±2.93	**0.11**	**0.03**	0.31
	**T**_**2**_	57.87±3.87	51.44±1.59	57.66±2.58			
	**T**_**3**_	48±4.14	51.72±1.39	52±3.87			
**LDL/HDL**	**T**_**1**_	1.96±3.8	2.3±1.5	2.26±2.87	**<0.001**	**<0.001**	**<0.001**
	**T**_**2**_	2.6±4.06	2.4±1.56	2.14±2.53			
	**T**_**3**_	4.81±5.37	2.26±1.36	2 ±3.8			
**TC (mg/dl)**	**T**_**1**_	201.5±26.78	203.39±10.6	211.84±21.01	**0.04**	**0.03**	0.19
	**T**_**2**_	210.57±28.63	219.17±11.05	223.27±17.85			
	**T**_**3**_	299.25±37.88	204.17±9.62	201.62±26.78			
**TG (mg/dl)**	**T**_**1**_	178.12±41.03	185.77±16.57	162.64±31.01	**0.004**	**0.004**	0.24
	**T**_**2**_	193.28±43.86	186.08±17.30	185.16±27.35			
	**T**_**3**_	381.75±58.02	173.19±14.85	180.87±41.03			

Mean values of cardiovascular risk factors across CETP genotypes (B1B1, B1B2, B2B2) based on tertiles of NEAP intake

*P*_*1*_ *P*-value for curd model, *P*_*2*_ *P*-value for the adjusted model by age, gender, physical activity, smoking, alcohol consumption, and familial history of diabetes

**Table 3:** The interaction between CETP Taq1B polymorphism and PRAL on cardiovascular risk factors

**Variables**	**DIL**	**Mean±SD**			**P**_**1**_	**P**_**2**_	
		**B1B1**	**B1B2**	**B2B2**	**Model (1)**	**Model (2)**	**P -trend**
**LDL (mg/dl)**	**T**_**1**_	108.1±10.52	123.85±4.8	128.53±8.59	**0.009**	**0.009**	**0.02**
	**T**_**2**_	121.6±14.88	118.43±4.57	120.68±8.31			
	**T**_**3**_	146.75±16.63	113.11±4.33	104.7±10.52			
**HDL (mg/dl)**		64.25±5.46	54.16±1.57	55.8±2.82	**0.02**	**0.01**	0.16
	**T**_**1**_						
	**T**_**2**_	56 ±4.88	50.18±1.5	60.87±2.73			
	**T**_**3**_	51.9±3.45	51.89±1.42	50.8±3.45			
**LDL/HDL**	**T**_**1**_	2.26±3.39	2.31±1.54	2.38±2.77	**<0.001**	**<0.001**	**0.001**
	**T**_**2**_	2.26±4.7	2.37±1.47	2.03±2.68			
	**T**_**3**_	4.81±5.36	2.26±1.39	2.09±3.39			
**TC (mg/dl)**	**T**_**1**_	196.1±23.97	206.83±10.94	217.85±20.26	**0.02**	**0.04**	0.19
	**T**_**2**_	225±33.9	213.6±10.41	208.5±18.95			
	**T**_**3**_	299.25±37.9	204.81±9.86	216.9±23.97			
**TG (mg/dl)**	**T**_**1**_	155.6±36.46	194.8±17	188.33±29.77	**0.001**	**0.001**	**0.04**
	**T**_**2**_	244.4±51.57	174.23±16.14	155.43±28.82			
	**T**_**3**_	381.75±57.65	175.77±15.14	180.3±36.46			

Mean values of cardiovascular risk factors across CETP genotypes (B1B1, B1B2, B2B2) based on tertiles of PRAL intake

*P*_*1*_ *P*-value for curd model, *P*_*2*_ *P*-value for the adjusted model by age, gender, physical activity, smoking, alcohol consumption, and familial history of diabetes

**Fig. 1:**
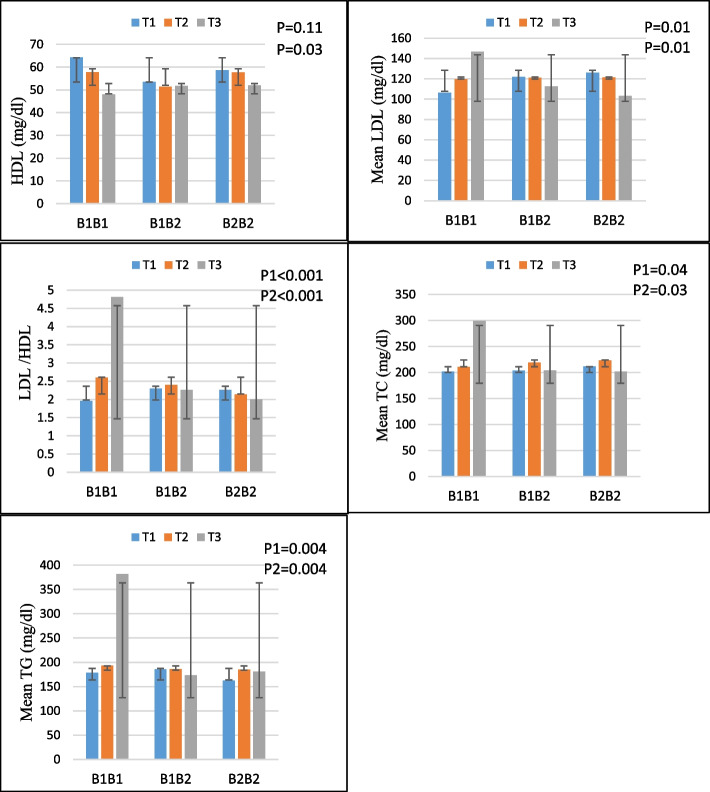
Interaction effect between NEAP (mEq/d) and CETP on (**a**): HDL, (**b**): LDL, (**c**): LDL/HDL, (**d**): TC, (**e**): TG **P **_**1**_ = *P* value with unadjusted (crude) model, **P **_**2**_ = *P* value with adjustments for potential confounding factors including (age, physical activity, sex, smoking, alcohol and energy intake)

In terms of lipid profiles including (HDL, LDL, TC, LDL/HDL, and TG) in both crude and adjusted models, there were significant interactions between NEAP and PRAL scores and CETP Taq1B. This study found that individuals with the B1B1 genotype had higher LDL (P1-interaction = 0.01, P2-interaction = 0.01), TC (P1-interaction = 0.04, P2-interaction = 0.03), LDL/HDL (P1-interaction 0.001, P2-interaction 0.001), and TG (P1-interaction = 0.004, P2-interaction = 0.004) when they consumed diets that scored higher on the NEAP index. Furthermore, the adjustment model revealed a significant rs708272-NEAP interaction on HDL concentration (P Interaction = 0.03), and B1B1 allele carriers with the highest tertile of NEAP had lower HDL levels (Fig. 1).

**Fig. 2:**
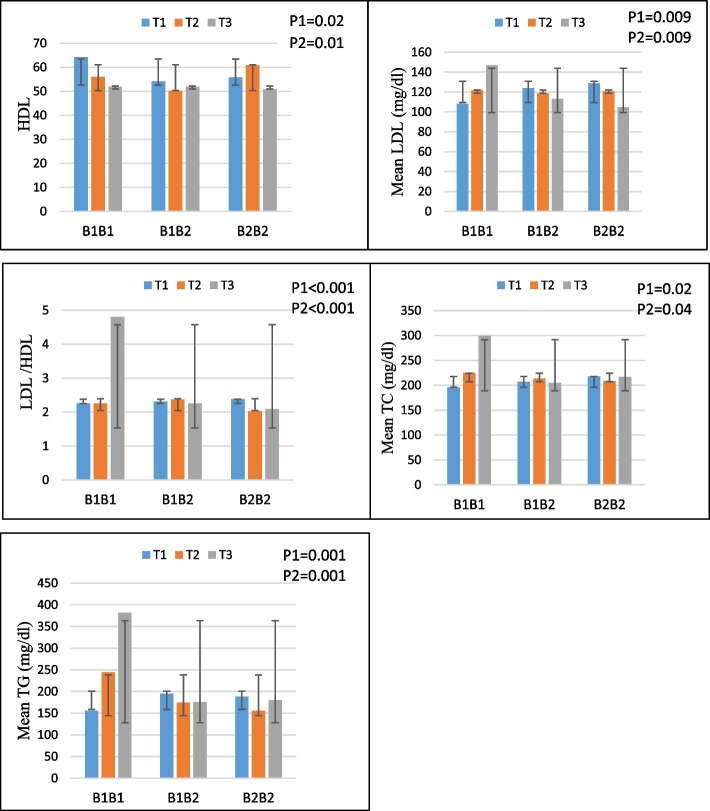
Interaction effect between PRAL (mEq/d) and CETP on (**a**): HDL, (**b**): LDL, (**c**): LDL/HDL, (**d**): TC, (**e**): TG **P **_**1**_ = *P* value with unadjusted (crude) model, **P **_**2**_ = *P* value with adjustments for potential confounding factors including (age, physical activity, sex, smoking, alcohol and energy intake)

The highest tertiles of the PRAL also showed higher levels of TG (P1-interaction = 0.001, P2-interaction = 0.001), LDL (P1-interaction = 0.009, P2-interaction = 0.009), TC (P1-interaction = 0.02, P2-interaction = 0.04), LDL/HDL (P1-interaction 0.001, P2-interaction 0.001), and TG (P1-interaction = 0.001, P2-interaction. Lower HDL concentration was seen in those with B1B1 genotypes in the upper tertile of PRAL (P1-interaction = 0.02, P2-interaction = 0.01) (Fig. 2).

## Discussion

So far as we know, the effect of the interaction between CETP *Taq1B* and DAL on lipid profile was evaluated in this study for the first time. Here, the protein, carbohydrate, and cholesterol intake increased significantly by the adherence to DAL indexes (PRAL and NEAP). In the present study, not only did lipid profile have insignificant differences through tertiles of indexes, but also in genotypes. In agreement with the findings, various studies have noted a non-significant association between DAL indexes, genotypes, and lipid profile [10, 32, 40–47] whereas several reported either a direct or an inverse link between them [32, 42, 48–51]. Changes in lipid profile in relation to DAL might be interpreted by an increase in the production of cortisol which is affected by metabolic acidosis provoked by DAL. In this case, the lipase activity is aroused which leads to an intensifying production of very-low-density lipoproteins (rich in TG) [52, 53]. Employing the polymorphism, a greater level of HDL-C in *B2B2* homozygotes has been indicated in the findings of the present study as well as some others despite whether the association between lipid profile and genotypes is significant. It’s important to underline that the other polymorphism in the region of the *Taq1b* might also affect the HDL-C level as well as the above-mentioned polymorphism [44, 54–56]. Still, the differences in health status, dietary assessment tool, sample size, ethnicity, age, and type of population participating in various studies should be taken into account for discussing the different findings with respect to the actual relationship between DAL, genotypes, and lipid profile.

The key finding of the present study was an increased level of LDL-C, TC, TG, and LDL-C/HDL-C ratio while HDL-C concentration dropped through the interaction between CETP *Taq1B*, NEAP, and PRAL, notably in *B1B1* homozygotes (wild type) despite neither lipid profile nor indexes scores were statistically different in genotypes. Much less effort has been made in studying the polymorphism-dietary patterns interaction while many studies have investigated either the linkage of the CETP polymorphism and lipid profile or the interaction between genotypes and dietary components on lipid profile. Concerning this, Kalantar et al. [14, 47] reported that total fat intake modifies the association of CETP *Taq1B* polymorphism and HDL-C level in normolipidemic T2DM participants and *B1B1* subjects are prone to have a decreased LDL/HDL ratio by the adherence to healthy eating index, dietary quality index, and dietary phytochemical index. Through the study of Abaj et al. [57], a greater following of dietary insulin index and dietary insulin load in T2DM patients was related to a lower level of TG, LDL/HDL ratio, and a higher level of HDL-C in *B1B1* homozygotes. By the report of Campos-Perez et al. [58], subjects with the *B1B2/B2B2* genotypes had elevated levels of TC and LDL-C by more sucrose intake. As stated by Gammon et al. [59], an improvement in TG/HDL-C ratio was observed by consuming two green kiwifruits a day along with a healthy dietary pattern in *B1B1* homozygotes. An elevated level of HDL-C after consuming a high carbohydrate and low fat (HC/LF) diet was linked with the *B2* allele by the findings of Du et al. [60] while men with *B1B1* genotypes were more vulnerable to getting affected by HC/LF diet on their HDL-C concentration. Though the absolute underlying mechanism of the aforementioned relationship is still vague, some conceivable clarification might be available. The *B1B1* subjects of the present study consuming fewer amounts of fruits and vegetables as opposed to *B2B2* homozygotes, resulting in a lower intake of potassium, however, the scores of the indexes did not differ between genotypes. It is proposed that consuming high amounts of fruits and vegetables could drop DAL by various justifications [61]. The acidifying effect of vegetable proteins is not comparing the same as animal proteins and their phosphorus content is less bioavailable by the form of phytate [62]. Moreover, the metabolism of the potassium salts i.e. malate and citrate lead to the consumption of hydrogen ions which might give vegetables and fruits a neutral effect on the acid load and accordingly, they’re considered as sources of alkalizing foods which have negative effects on DAL indexes [62–64]. Besides, the accessibility of dietary fat could be limited via dietary fiber through fat absorption reduction which could lessen hepatic synthesis of cholesterol while binding to bile acid which facilitates lipid circulation modification [65–68]. Dietary fiber could additionally control the metabolic activity and metabolites of the intestinal flora [69] which could be another explanation for the elevated level of lipid profile in *B1B1* genotypes with low consumption of vegetables and fruits, and high adherence to DAL. At the same time, the CETP *Taq1B* polymorphism is linked with the serum lipid profile that codes the CETP protein which is responsible for the reverse transfer of cholesterol ester. In this regard, it has been proposed that the *B1* allele has been linked with the risk of dyslipidemia due to the linkage with further CETP activity and increased TG. On the other hand, the protective one (*B2* allele) is related to a lower level of serum CETP which decreases the reverse transfer of cholesterol ester and carries out an increase in HDL-C level [18, 70, 71], yet, more investigations are required to provide insight into the interaction effect of the CETP *Taq1b* and DAL on lipid profile.

Provided the novelty of the study, still it has some limitations. Above all, it’s a cross-sectional study without measuring CETP concentration across genotypes and no causality was provided about detected interactions. Recall bias and over-or under-reporting of the dietary intake were other limitations of the present study which might happen by using the FFQ.

## Conclusion

Conclusively, the amount of LDL-C, TC, TG, and LDL-C/HDL-C ratio elevated while HDL-C concentration dropped in *B1B1* homozygotes by greater adherence to NEAP and PRAL which might be the result of consuming more fruits and vegetables. The findings could be utilized together with the genetic relation of the patients to yield appropriate nutritional advice to prevent or amend the cardiovascular risk factor of type 2 diabetes, nevertheless, it should be confirmed in high-quality interventional studies.

### Comparisons with other studies and what does the current work add to the existing knowledge

The identification of the aforementioned interactions could be used in personalized nutritional recommendations for the refinement and prevention of type 2 diabetes complications and for ameliorating the lipid profile of T2DM patients.

## Strengths

This is the first study attempt to explore the interaction effect of the DAL and CETP *Taq1b* polymorphism on lipid profile in type 2 diabetic patients.

## Limitations

It is a cross-sectional study without measuring CETP concentration across genotypes and providing any causality about detected interactions. Recall bias and over-or under-reporting of the dietary intake was another limitation of the present study which might happen by using the FFQ.

## Data Availability

The corresponding author will provide the datasets used and/or analyzed during the current work upon reasonable request.
